# Management of Unruptured Traumatic Middle Meningeal Artery Pseudoaneurysms through Onyx Embolization

**DOI:** 10.7759/cureus.1794

**Published:** 2017-10-23

**Authors:** Nicolas K Khattar, Andrew C White, Enzo M Fortuny, Rob T Hruska, Robert F James

**Affiliations:** 1 Neurological Surgery, University of Louisville School of Medicine; 2 Radiology, University of Louisville School of Medicine

**Keywords:** middle meningeal artery, pseudoaneurysm, embolization, onyx, trauma

## Abstract

Traumatic pseudoaneurysms of the middle meningeal artery (MMA) represent less than 1% of all intracranial aneurysms, and occur mostly in association with temporal bone fractures following head trauma. Given the unknown natural history, it is unclear whether they should be treated. We present two cases of MMA pseudoaneurysms discovered during trauma workups. The first patient is a 44-year-old male with severe traumatic brain injury (TBI) following a motor vehicle accident. The patient was found to have two right-sided middle meningeal artery pseudoaneurysms that were treated successfully with Onyx® (Medtronic, Minneapolis, MN) embolization. The second patient is a 56-year-old male that sustained a severe TBI and skull fracture following a motorcycle collision. Angiography demonstrated an unruptured right MMA aneurysm, which was also treated successfully with Onyx embolization. MMA pseudoaneurysms occur rarely in the setting of severe traumatic injuries. In select patients, treatment by an experienced neuro-interventionalist can prevent highly morbid future intracranial hemorrhages with minimal risk of complications.

## Introduction

Traumatic pseudoaneurysms of the middle meningeal artery (MMA) are rare and potentially serious complications of traumatic brain injuries [1]. Delayed epidural hematoma is the most common manifestation of these lesions, as such aneurysms are often radiographically occult during the initial trauma evaluation [2, 3]. Occasionally, traumatic pseudoaneurysms are discovered incidentally on neuroimaging as part of the initial workup [2]. The natural course of these aneurysms is largely unknown: there have been reports of spontaneous resolution, as well as reports of aneurysm growth and subsequent rupture [2-4]. The decision to intervene needs to be carefully weighed against the risk of peri-procedural morbidity and mortality [1, 4]. Endovascular embolization techniques have significantly progressed in recent years and are a safe and effective treatment of cerebral aneurysms, whether intracranial or extracranial. In this report, we present two cases of unruptured traumatic MMA pseudoaneurysms successfully treated endovascularly via Onyx® (Medtronic, Minneapolis, MN) embolization.

## Case presentation

The first patient is a 44-year-old male, involved in a motor vehicle accident, who sustained a severe traumatic brain injury resulting in an acute subdural hematoma, intraparenchymal and subarachnoid hemorrhages. An emergent decompressive craniotomy was performed at a community hospital and no specific vascular lesions of the MMA were appreciated intra-operatively. Consequently, the patient was referred to our tertiary care center for evaluation of vascular injuries discovered on computed tomography (CT)e angiography. Cerebral angiography revealed two traumatic pseudoaneurysms of the right MMA. The decision was made to treat the MMA aneurysms using endovascular Onyx 18 embolization per standard technique, with excellent results. 

**Figure 1:**
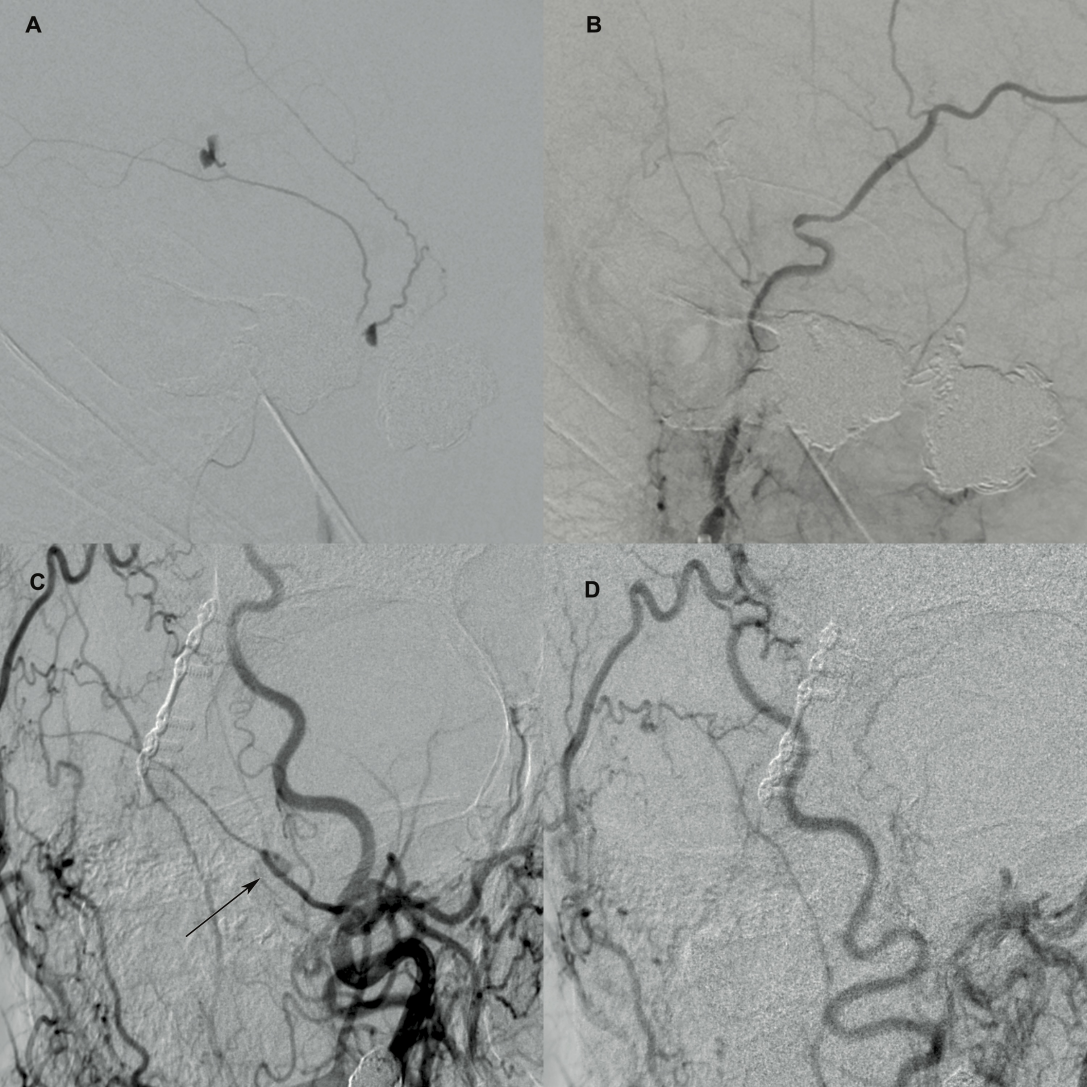
Cerebral angiography showing the traumatic pseudo-aneurysm pre- and post-treatment A: Initial external carotid artery injection showing the two unruptured middle meningeal artery traumatic pseudoaneurysms of patient one  B: Follow-up angiography showing persistent complete resolution  C: External carotid artery injection showing the saccular traumatic pseudoaneurysm of the middle meningeal artery of patient (arrow) two  D: Complete resolution of the aneurysm following Onyx embolization

The second patient is a 56-year-old male that sustained a frontal bone fracture with severe cerebral edema following a motorcycle collision. Field resuscitation and intubation were completed prior to arrival to the emergency department. Initial survey showed a large scalp laceration, periorbital ecchymosis, scattered chest and abdominal abrasions, multiple orthopedic fractures, which required surgical fixation as well as extensive intra-abdominal injuries, which required a laparotomy and repair. An external ventriculostomy drain was placed for cerebrospinal fluid diversion and intracranial pressure monitoring. Critical care management was done for eight days and the patient was extubated. Follow-up CT Angiography showed bilateral carotid artery dissection and various other traumatic vascular lesions that were treated according to standard guidelines. Follow-up angiography performed one year later revealed a right-sided traumatic unruptured middle meningeal artery aneurysm in addition to persistent right-sided internal carotid artery (ICA) dissection with an enlarging pseudoaneurysm. The decision was made to treat both lesions concurrently. The right MMA aneurysm was treated with transcatheter embolization using Onyx 34, with excellent angiographic results. The ICA pseudoaneurysm was treated using overlapping pipeline embolization. Figure 1 shows the pre- and post-intervention cerebral angiograms.

Neither patient experienced any intraoperative or immediate postoperative complications. The first patient remained mechanically ventilated in the perioperative period and his respiratory status steadily improved. He was discharged several days later to an acute rehabilitation facility. There was no recurrence of any of the lesions at 10-month follow-up angiography. The second patient tolerated the procedure well and was discharged home the next day in stable condition. Follow-up angiography one year later showed no recurrence of lesions post-embolization.

## Discussion

Pseudoaneurysms of the middle meningeal artery are a rare complication of severe traumatic brain injury, where 70-90% of all cases occur in the setting of a temporal bone fracture along the course of the artery. The remainder of the cases occurs in the setting of severe traumatic brain injury independent of fracture sites, or as iatrogenic lesions [2]. True cerebral aneurysms of the MMA contain all histologic vascular layers and are associated with non-traumatic conditions associated with increased hemodynamic stress or specific diseases of the MMA [5]. Pseudoaneurysms of the MMA are a separate entity and are often identified as a cause of delayed intracranial hemorrhage, the most frequent manifestation of which is an epidural hematoma. Given the low incidence of these lesions, there is no consensus regarding the management of incidental unruptured MMA pseudoaneurysms. The risk profile of any intervention needs to be carefully evaluated in trauma patients, who can be in critical condition due to the severity of the initial trauma [6]. Some reports have shown that pseudoaneurysms exhibit an accelerated rate of growth and, thus, portend a greater risk of rupture, which approaches 20% [7, 8]. If left untreated, patients are at risk of developing an acute or delayed hemorrhage, which could be epidural, intra-parenchymal or subarachnoid in nature [5, 6, 9]. Traditionally, management of these traumatic aneurysms is conservative, with craniotomy and electrocautery of the vessel or microsurgical clip ligation being reserved for the event of a spontaneous rupture.

In the past decade, endovascular therapy has emerged as a safe and effective alternative to open surgery. Various endovascular techniques have been successfully used to treat traumatic pseudoaneurysms including coiling, liquid embolization, and stenting [1, 10]. Embolization in the external carotid circulation generally carries a lower risk profile than procedures performed in the internal carotid circulation. Meningiomas and dural arteriovenous fistulas supplied by a middle meningeal artery are commonly embolized lesions with a low risk of stroke or intracranial hemorrhage. In our series, we present two cases where liquid embolization with Onyx was safely and successfully used to treat the pseudoaneurysms with no recurrence on follow-up angiography. Unruptured pseudoaneurysms may cause high morbidity or mortality in the event of a spontaneous rupture. Even though our series includes only two patients, early embolization of these traumatic lesions is favorable given the efficacy and low-risk profile of endovascular management.

## Conclusions

Unruptured traumatic pseudoaneurysms of the middle meningeal artery are rare but potentially devastating lesions associated with severe traumatic brain injury. Although their natural history is largely unknown, treatment of these aneurysms should be considered for the prevention of severe morbidity and mortality associated with their potential rupture. When performed by an experienced neuro-interventionalist, Onyx embolization provides a safe, effective, and durable treatment option for these lesions.
